# Gallbladder cancer: review of a rare orphan gastrointestinal cancer with a focus on populations of New Mexico

**DOI:** 10.1186/s12885-018-4575-3

**Published:** 2018-06-18

**Authors:** Jacklyn M. Nemunaitis, Ursa Brown-Glabeman, Heloisa Soares, Jessica Belmonte, Ben Liem, Itzhak Nir, Victor Phuoc, Rama R. Gullapalli

**Affiliations:** 1 Comprehensive Cancer Center, University of New Mexico, Albuquerque, NM USA; 2 Department of Internal Medicine, Division of Hematology and Oncology, University of New Mexico Health Sciences Center, Albuquerque, NM USA; 3 Department of Surgery, Division of Surgical Oncology, University of New Mexico Health Sciences Center, Albuquerque, NM USA; 4 Department of Pathology, University of New Mexico Health Sciences Center, Albuquerque, NM USA; 5 Department of Chemical and Biological Engineering, University of New Mexico, Room 333A, MSC08-4640, Albuquerque, NM 87131 USA

**Keywords:** Gallbladder Cancer, New Mexico, Gallstones, Personalized medicine, *HER2/Neu*, Chronic Inflammation, Heavy Metals

## Abstract

Gallbladder cancer is a rare malignancy of the biliary tract with a poor prognosis, frequently presenting at an advanced stage. While rare in the United States overall, gallbladder cancer has an elevated incidence in geographically distinct locations of the globe including Chile, North India, Korea, Japan and the state of New Mexico in the United States. People with Native American ancestry have a much elevated incidence of gallbladder cancer compared to Hispanic and non-Hispanic white populations of New Mexico. Gallbladder cancer is also one of the few bi-gendered cancers with an elevated female incidence compared to men. Similar to other gastrointestinal cancers, gallbladder cancer etiology is likely multi-factorial involving a combination of genomic, immunological, and environmental factors. Understanding the interplay of these unique epidemiological factors is crucial in improving the prevention, early detection, and treatment of this lethal disease. Previous studies have failed to identify a distinct genomic mutational profile in gallbladder cancers, however, work to identify promising clinically actionable targets is this form of cancer is ongoing. Examples include, interest in the *HER2*/*Neu* signaling pathway and the recognition that chronic inflammation plays a crucial role in gallbladder cancer pathogenesis. In this review, we provide a comprehensive overview of gallbladder cancer epidemiology, risk factors, pathogenesis, and treatment with a specific focus on the rural and Native American populations of New Mexico. We conclude this review by discussing future research directions with the goal of improving clinical outcomes for patients of this lethal malignancy.

## Background

Gallbladder cancer (GBC) is a rare malignant neoplasm [1]. It is, however, the most common malignancy of the biliary tract and sixth in overall gastrointestinal cancer incidence [1, 2]. GBC is an aggressive, often lethal malignancy with a mean overall survival of six months [1, 2]. The disease is rapidly progressive and rarely diagnosed at an early stage, contributing to poor clinical outcomes.

The worldwide incidence of GBC is less than 2/100,000 individuals and there is considerable variation in this number based on geographic distribution and gender [3]. This variability is likely due to a combination of environmental and genetic factors. Women have a higher incidence ratio (F:M ratio ~ 2.6:1), with typical onset in the sixth decade. Inordinately high rates of GBC occur among Native Americans and Southeast Asians, with several other scattered geographical locations of elevated GBC incidence across the world [1, 4, 5]. While the disease is rare overall in the United States, Native Americans in New Mexico have uniquely high rates of GBC [4]. Native American women are more commonly affected compared to Native American men, in line with globally observed trends [4].

Due to the rarity of this cancer, GBC is an orphan disease with minimal research efforts currently (both basic and translational) in the United States. Currently, no targeted therapies exist for the treatment of GBC in routine clinical practice [6]. A better understanding of GBC pathogenesis is urgently needed to develop targeted therapy approaches to improve outcomes in GBC patients. In this review, we discuss the pathogenesis of GBC, the current status of GBC therapies, the future directions with a focus on GBC pathogenesis in rural and Native American populations of New Mexico.

## Epidemiology and risk factors for gallbladder cancer

The geographic distribution of GBC incidence is highly uneven, with remarkable rarity in certain parts of the world, and near epidemic rates in others [5]. This uneven incidence distribution is likely due to a combination of genetic and local environmental factors. The highest rates of GBC are found in Latin America and Asia and the lowest rates are seen in the United States and parts of Western and Mediterranean European countries [7] . For example, among Chilean women, GBC is the leading cause of cancer death, with mortality rates higher than those for breast or lung [3, 5]. Other high-risk areas around the world include Poland (14/100,000), northern India (21.5/100,000 for women), south Pakistan (11.3/100,000), Israel (5/100,000), and Japan (7/100,000) [1].

Interestingly, the highest rates of GBC are generally found in indigenous Amerindian populations of the Americas (e.g., Chile, Bolivia, and states such as New Mexico in the United States). Among Native Americans in the United States (including Alaska Natives) the rate of GBC incidence is 3.3/100,000 [8] compared to the 0.4–1.5/100,000 incidence rate seen in the non-Native American populations. Native Americans from the state of New Mexico have the highest rates of GBC incidence in the United States [4]. The elevated GBC incidence is observed in Native American women and men compared to the other ethnicities in New Mexico (Caucasians and Hispanics in New Mexico) [4]. Interestingly, the Hispanic populations of New Mexico also show an elevated incidence of GBC (compared to Caucasians in New Mexico), though not to the degree seen in Native Americans [4]. In Chile, for each 1% increase in Mapuche heritage, an indigenous people common in Chile, there is an associated 3.7% increase in risk for GBC [9]. These observations argue for specific predisposing genetic factors (direct or indirect) unique to certain indigenous populations.

Numerous risk factors driving GBC have been studied including demographic factors, pre-existing gallbladder disease, and environmental exposures. Predisposing conditions for GBC include chronic gallstones [10], chronic infections leading to cholangitis (e.g., Salmonella enterica typhi and *H. pylori* seen mainly in India and Bangladesh) [11–14], porcelain gallbladder [15], Mirizzi’s syndrome [16], bile reflux [17], and gallbladder polyps. Environmental factors such as diet, toxic ingestions, aflatoxins, elevated capsaicin consumption and vitamin deficiencies have all been controversially linked to GBC pathogenesis [1, 6, 13, 18, 19]. Obesity and poor socio-economic conditions are associated with elevated GBC incidence, though the underlying mechanisms remain uncertain.

The underlying causes of the elevated female incidence of GBC is unknown. Endogenous estrogen plays a key role in gallstone development [20]. Prolonged lifetime estrogen exposure is associated with elevated risk of GBC carcinogenesis [21, 22] through low parity, young age at menarche, late age at first pregnancy, oral contraceptive use, estrogen replacement therapy, and prolonged fertility [20]. A recent meta-analysis of 14 studies found an increased risk of GBC in overweight women (relative risk 1.25), however, a similar relationship was not identified in overweight men [23]. This correlation is interesting given the well-established link between obesity in women and elevated estrogen levels. Definitive biological mechanisms of the numerous risk factors associated with GBC carcinogenesis are lacking currently. This is mainly due to the paucity of readily available cell and animal models of GBC carcinogenesis which remain to be developed.

## Gallbladder cancer in New Mexico

**Fig. 1:**
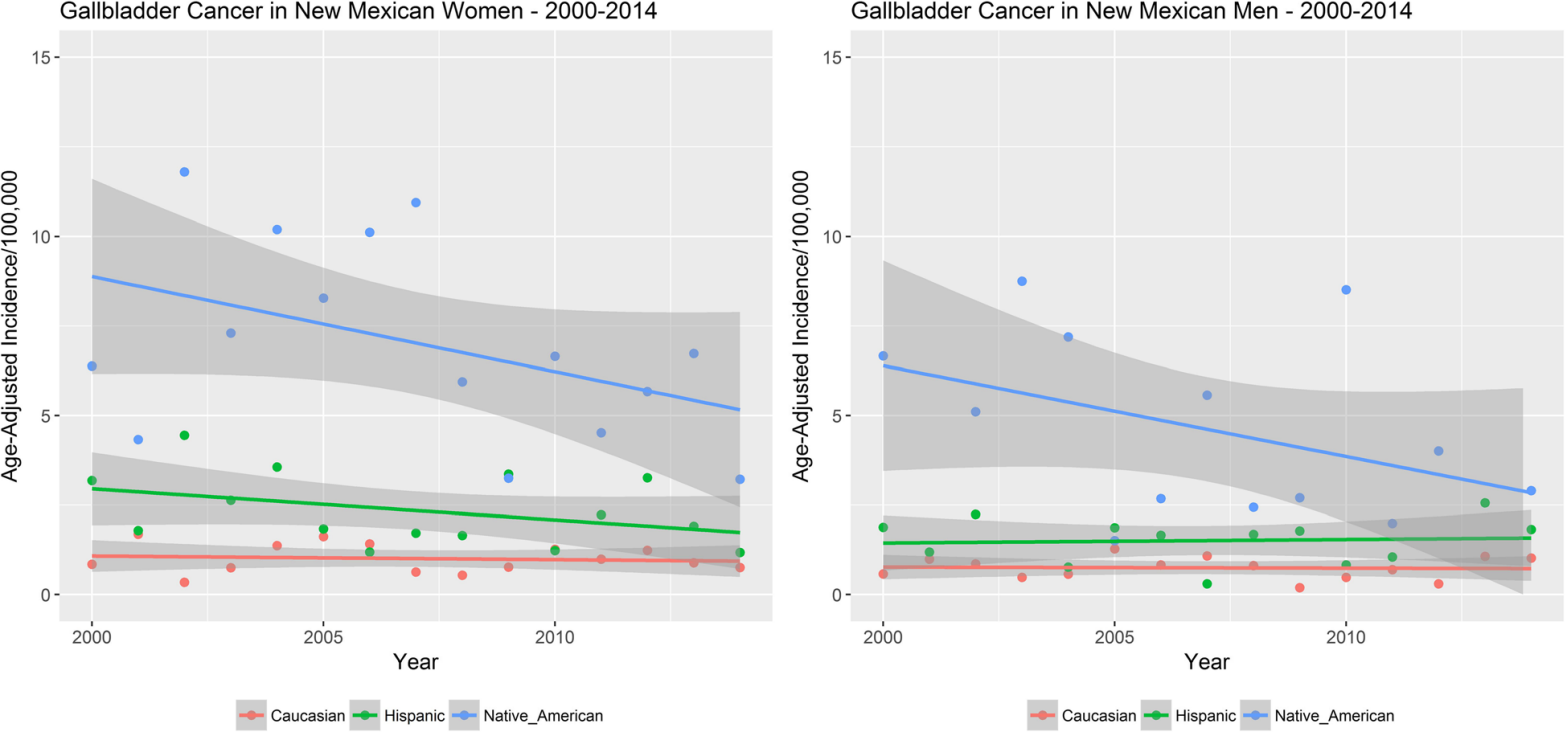
Age-adjusted GBC incidence among women (left) and men (right) of New Mexico from 2000 to 2014. Native American women and men show highly elevated incidence of GBC relative to Hispanics and Caucasians of New Mexico. The wide variation (gray) seen in Native American GBC incidence is due to the smaller population size and the number of annual GBC cases relative to Hispanics and Caucasians of New Mexico. Data is age-adjusted to the 2000 U.S standard population

Native American populations of New Mexico have a significantly elevated incidence of GBC compared to Hispanic and non-Hispanic white populations within the state [4]. The state of New Mexico is ethnically diverse with 45% non-Hispanic whites, 42% Hispanics and 10% American Indians. Nir et al. reviewed the SEER cancer registry data from 1981 to 2008 to identify trends of biliary tract cancer incidence (GBC and biliary tract cancers) in New Mexico [4]. Similar to epidemiological patterns seen elsewhere in the world, Nir et al. observed GBC was the commonest malignancy in the biliary tract with a higher incidence among women compared to men across all ethnicities in New Mexico. Native American women showed an eight fold higher GBC incidence compared to non-Hispanic whites while a four fold higher incidence was seen among Hispanic women compared to Caucasian women [4]. Among men, the GBC incidence among the Native Americans, Hispanic and Caucasian ethnicities was 4.1, 1.1 and 0.8% respectively [4]. We reviewed the publicly available GBC incidence data for this review paper from the New Mexico tumor registry (https://www.cancer-rates.info/nm/) for the years 2000–2014 (see Fig. 1). Consistent with the data from Nir et al. [4], we continue to observe an elevated incidence of GBC among Native American minorities relative to the Caucasians and Hispanics within the state of New Mexico from 2000 to 2014. The elevated GBC incidence trend is seen in both Native American men and women of the state (Fig. 1). The age adjusted incidence rate of GBC in New Mexico was 6.52, 2.17, and 0.96 per 100,000 among Native American, Hispanic, and Caucasian women respectively. For men, similar age-adjusted GBC incidence rates were 3.73, 1.27 and 0.71 per 100,000 (Native American, Hispanic, and Caucasian).

An elevated prevalence of gallstones amongst Native Americans may be one of the key risk factors associated with the increased GBC development. A prolonged clinical history of gallstones is the single most important risk factor associated with the development of GBC. The Native American populations of the American continent have a unique association with increased gallstone incidence [24]. Gallstone disease was identified as “the native man’s burden” by researchers as early as 1960s [25]. Sievers and Marquis first identified the elevated incidence of gallstones among the Native American populations of the South-Western United States, including the Navajo, Zuni and Hopi populations of New Mexico and Arizona [26]. It is now recognized that the incidence of gallstones is high among Native American populations across the entire American continent [24, 25, 27]. Canadian Indians have a 62% estimated incidence of gallstones. The prevalence of gallstones is approximately 70–80% among Pima Indian women of the age 25–35 years [25]. Similar patterns of elevated incidence are seen in Native American populations of South America (e.g., Mapuche Indians). In contrast, incidence of gallstones among non-Hispanic white women and men is reported to be 16.6 and 8.6% respectively [4].

Mortality from GBC in the past few decades has declined in part due to increased surgical intervention for gallstone removal. From 1970 to 1980, the mortality of GBC declined sharply in countries that performed more frequent cholecystectomies. In developed countries including England, Scotland, Canada, United States and Sweden, mortality from GBC is inversely associated with the frequency of cholecystectomy [10]. Alongside the modern plateau in surgical intervention for gallstones, incidence rates of GBC in the entire United States has remained stable [28]. However, in New Mexico the incidence of GBC amongst all ethnic groups, including Native Americans, appears to be gradually declining [4]. Gallstones in the developed world (including the Native Americans) tend to be of the cholesterol type reflecting the role of obesogenic diets [25]. However, Native Americans develop gallstones at an earlier age without a correlation to obesity suggesting a genetic basis of gallstone formation [25]. While the molecular genetics of gallstones have been studied in detail across the world, similar gene association studies in populations of New Mexico are lacking currently.

While gallstones are an important risk factor in GBC carcinogenesis, it does not fully explain the increased risk of GBC. The majority of patients (~ 98%) with gallstones never develop GBC suggesting the need for additional ‘hits’ in the GBC pathogenesis process. Various environmental risk factors have been examined in detail with no clear correlation at the current time [29]. It is very likely the second ‘hit’ in GBC are risk factors unique to the geographical locations (e.g., Salmonella in India, Aflatoxins in South America [18]). One under-explored hypothesis is the role of heavy metals in GBC pathogenesis. Basu et al. examined the presence of heavy and trace metals in samples of GBC from India [30] and identified significantly elevated copper concentrations in the serum, bile and gallbladder tissue samples of GBC patients [22]. Elevated levels of lead, cadmium, chromium and nickel levels were also seen in the serum and bile of these patients. A second study by Chhabra et al. identified elevated levels of chromium, lead and arsenic in Indian GBC patients compared to GBC patients from Japan [31]. The precise mechanisms by which heavy metals induce carcinogenic transformation in gallbladder remains unknown.

**Fig. 2:**
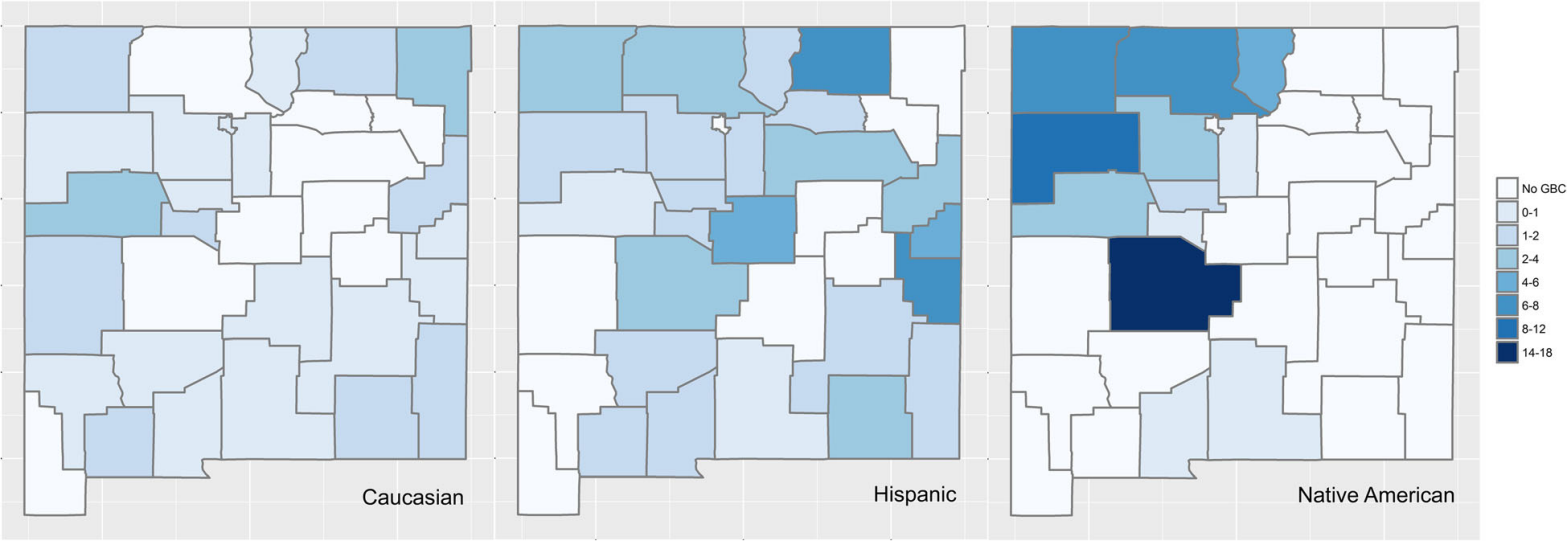
Age-adjusted GBC incidence data (2000–2014) mapped to the 33 counties of New Mexico. Darker color intensity represents elevated GBC incidence. Native American populations who live predominantly in the north-west portion of New Mexico (e.g., Navajo reservation) show the highest GBC incidence

The southwestern portion of United States has a legacy of abandoned mines, the most significant of which is Uranium mining in the Four Corners location of the Navajo Nation [32]. More than 10,000 abandoned mines currently exist in western United States in states of New Mexico, Arizona and Colorado [32]. Many Native Americans live close to these abandoned mines with high levels of heavy metal exposures such as uranium, arsenic and cadmium due to contaminated water supplies in these communities [32–34]. Uranium is a well-known nephrotoxin while arsenic tends to act as co-carcinogen for various other heavy metals [35, 36]. It is possible that heavy metals may act as a “second-hit” acting in synergy with gallstone induced inflammation to drive gallbladder carcinogenesis among Native American populations of New Mexico. Using the publicly available data from the New Mexico Tumor registry, we also mapped the age-adjusted GBC incidence data at a New Mexico county level (see Fig. 2). The geospatial mapping shown in Fig. 2 vividly illustrates the regional preponderance of GBC incidence patterns within the state of New Mexico. The north-west corner of the state is home to the Navajo reservation where a large majority of the New Mexican Native American population currently resides. Unsurprisingly, this region also shows the highest incidence rates of GBC cases in New Mexico (Fig. 2). The Hispanic population is more prevalent in the North East and East portions of the state (adjacent to Texas) with localized increases GBC cases in these areas of the state within that ethnicity. The Caucasian population shows a diffuse, low incidence distribution of GBC across the entire state of New Mexico as expected. There is an increased density of abandoned mines near the Native American reservations (in the north-west part of the state, data not shown) representing an intriguing hypothesis of heavy metal exposure as one of the possible factors driving the elevated GBC causation among Native Americans which needs further study. Studies are currently underway in our lab to examine these exact issues in detail.

## Chronic inflammation: The common link of gallbladder cancer pathogenesis

Extensive evidence supports the role played by chronic inflammation in carcinogenesis in general and in GBC specifically [37]. GBC is a “classic” example of cancer pathogenesis driven by chronic inflammation [38]. Chronic inflammation of the gallbladder may be due to pre-existing gallstones or infections (e.g., Salmonella). A higher level of the acute phase reactant c-reactive protein (CRP) is associated increased risk of GBC [39]. Chronic inflammation induced carcinogenesis involves recurrent cycles of epithelial damage and repair. Long-term chronic inflammation results in the sustained release of inflammatory mediators, such as cytokines, chemokines, and prostaglandins into the tissue microenvironment. Cytokine induced inflammation may result in activation of oncogenes and inactivation of tumor suppressor genes leading to cell transformation, proliferation, and inhibition of apoptosis [38, 40].

Multiple inflammatory effector molecules have been linked to the development of GBC. Tumor necrosis factor alpha (*TNF-a*), a major pro-inflammatory cytokine, shows increased expression within the gallbladder mucosa from hyperplasia to carcinoma [41]. The relative expression of TNF-alpha increases with tumor stage [41]. Similarly, the chemokine (CXC motif) ligand 12 and its receptor *CXCR4* have been shown to play a role in tumor survival and metastases in GBC [42]. *CXCL12* is largely expressed in fibroblasts and is involved in cell proliferation, migration, and invasion. Both *CXCL12* and *CXCR4* are overexpressed in GBC [42] and has been linked with poor prognosis [43]. Another implicated molecule is cyclooxygenase 2 (*COX-2*). This molecule converts arachidonic acid to prostaglandins, contributing to chronic inflammation. *COX-2* has been found to be overexpressed in 91.7% of GBCs, whereas it is present in only 28.6% of chronic cholecystitis cases comparatively [44]. In one study, a significant association between the toll-like receptor polymorphisms in the *TLR2* and *TLR4* genes was associated with GBC [45]. The link between innate immunity and GBC is interesting due to the possible key role played by chronic Salmonella infections and GBC in India. The toll-like receptor genes are key players in identifying lipopolysaccharide elements associated with gram negative bacterial infections. In addition to the innate immunity *TLR* genes, other significant immune molecule associations with GBC include *IL1B, IL10* and *CCR5* [46]*.*

Chronic inflammation, via various chemokines, can result in somatic mutations linked with carcinogenesis. Some examples of this process include, *TP53*, a classic tumor suppressor gene, is the most frequently identified mutation in GBC, with a mutation rate of over 50% of GBC cases, and clearly plays a role in tumorigenesis [47]. Alterations in TP53 protein expression have been found in histologically normal gallbladders with chronic cholecystitis and the frequency of mutations increases as the pathology progresses to a malignant state [48]. Polymorphisms in *IL-10* and *VEGF* genes have been found to be related to higher risk of GBC [49]. MicroRNAs play an important role in both inflammation and cancer. They can act as inflammatory mediators, oncogenes, or tumor suppressors depending on the surrounding environment [50]. Not surprisingly, expression levels of certain miRNA strands in vitro significantly enhanced GBC proliferation and invasion [51]. The strong associations discussed above argue that there may be a role for anti-inflammatory agents in the prevention or treatment of GBC [52, 53]. Population studies have indicated that the use of aspirin, an inhibitor of COX-2 protein, may reduce the risk of GBC (OR 0.37) [54]. Several in vitro studies have been performed attempting to treat GBC with various anti-inflammatory agents with promising results [52], but there have been no human trials to date.

## Molecular pathogenesis and genomics of gallbladder cancer

**Fig. 3:**
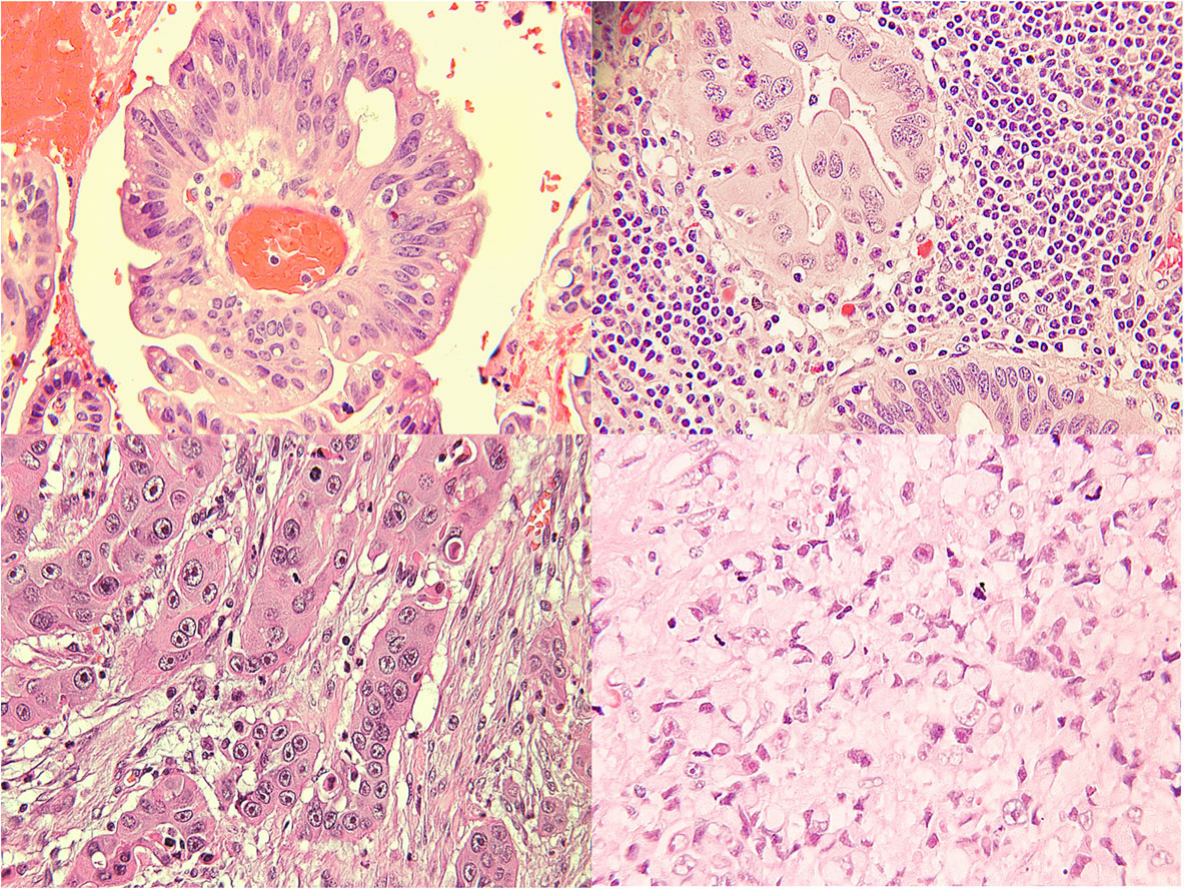
A hematoxylin and eosin stained image of gallbladder cancer histomorphology. The four panels (clockwise from top left) shows the differing degrees of differentiation commonly observed in gallbladder cancer pathology specimens (dysplasia, well differentiated, poorly differentiated, and moderately differentiated)

The pathological spectrum of progression in GBC involves various stages including metaplasia, dysplasia, carcinoma-in-situ and invasive cancer (Fig. 3) [6]. The most common pathway to GBC progression involves the intermediate stages of metaplasia and dysplasia. The classic adenoma-carcinoma sequence occurs in GBC with far less frequency (< 3%) [6]. The underlying somatic molecular changes of GBC remain ill-understood. However, based on previous studies we have a broad understanding of the commonly associated gene changes which are described below.

**Fig. 4:**
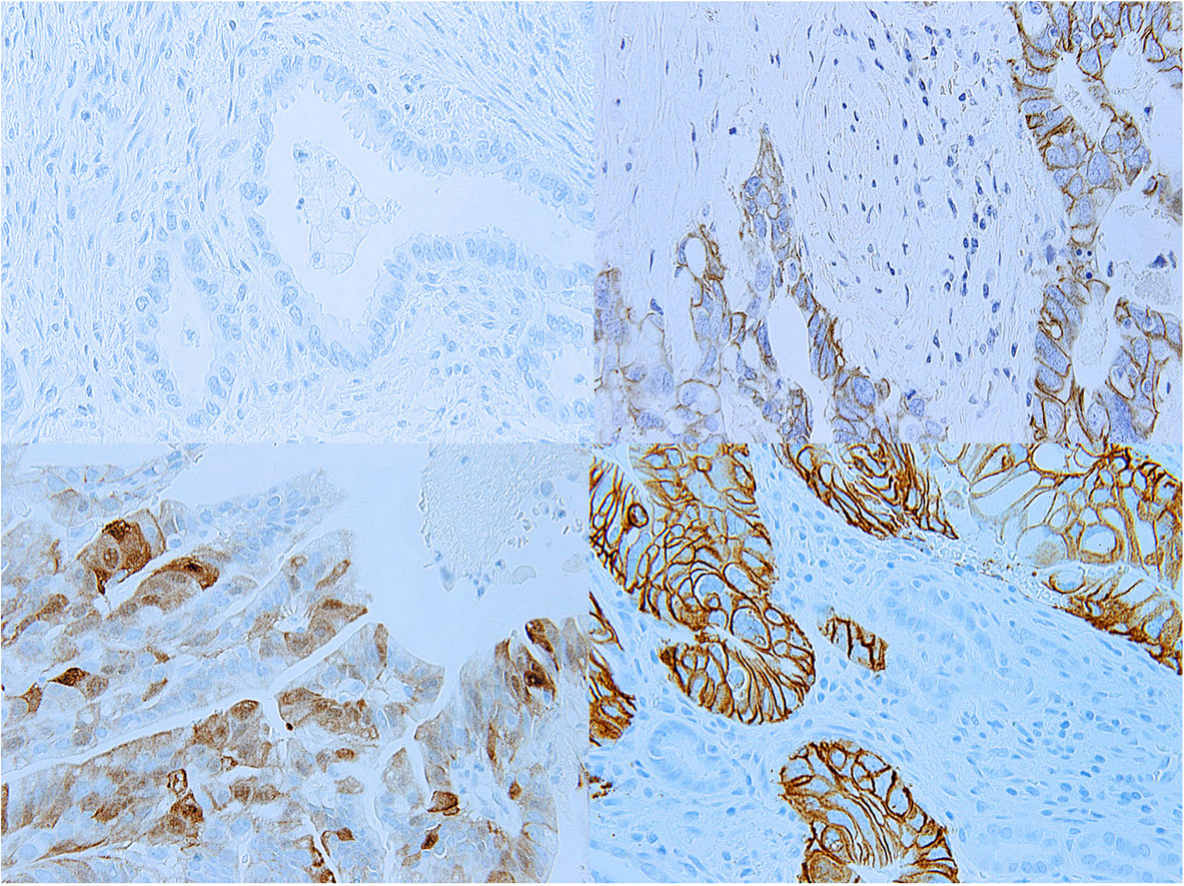
HER2 positivity in New Mexican cases of gallbladder cancer by immunohistochemistry. The panels (clockwise from top left) represent HER2 staining status of 0, 1+, 3+ and 2+ grades. Gallbladder cancer HER2 staining is axial, similar to the staining pattern seen in gastric cancers. In contrast, HER2 staining in breast cancers is more circumferential and evenly distributed around the tumor cell periphery

**Fig. 5:**
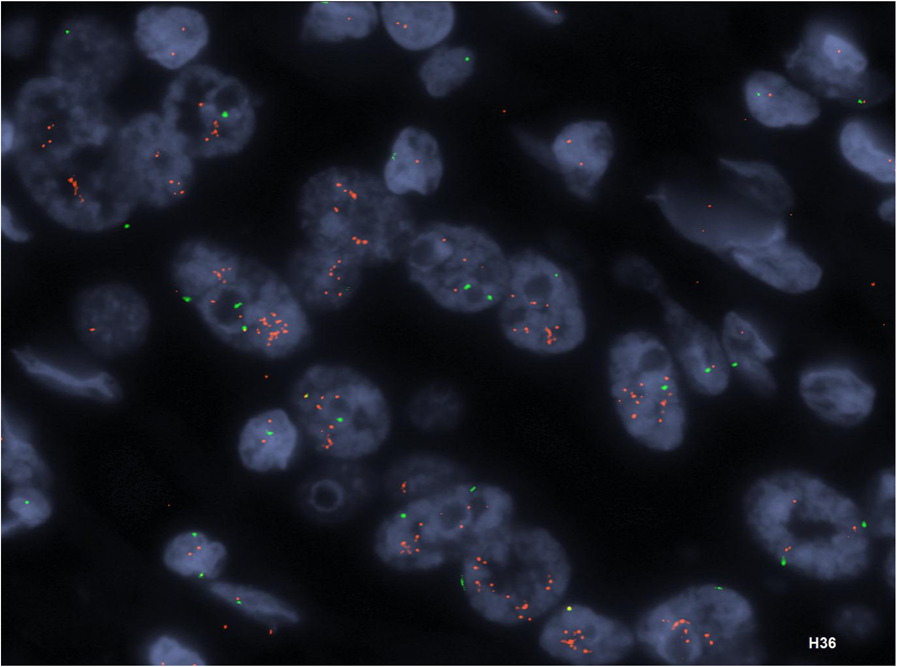
*HER2* gene amplification detected by Fluorescence In-Situ Hybridization (FISH) method. This case had approximately nine *HER2* gene copies per cell and showed a concordant 3+ staining for HER2 protein expression by IHC

- Somatic and copy number changes: A variety of genomic changes have been identified in GBC [6, 55]. These include somatic mutations, microsatellite instability, loss of heterozygosity, gene overexpression, epigenetic changes and miRNA associated changes [55]. Different cell signaling pathways are implicated in the causation of biliary tract cancers and GBC. These include the *ErbB* pathway [56, 57], *PI3K/Akt/mTOR* pathway [58], *MAPK/ERK* pathway [59], *VEGF* pathway [60], *Notch* pathway [61] and *Hedgehog* pathway [62] to name a few. GBC pathogenesis involves the complex interaction of various genomic changes at a DNA, RNA and epigenetic level. Li et al. performed the first comprehensive mutational profiling in GBC [63]. This study identified ~ 1450 somatic changes at an exome level. The most commonly mutated genes included the *TP53, KRAS*, and the *ErbB* pathway genes. An overrepresentation of somatic mutational alterations was seen in the *ErbB* signaling pathway, comprised of the *EGFR, HER2, ERBB3, and ERBB4* genes, was seen in this study (*n* = 21/57) [63]. *ErbB* pathway genes are key growth factor receptor genes commonly implicated in multiple types of cancers. In addition, gene amplification of *HER2* and overexpression of *EGFR* and *HER2* has been observed in GBC in multiple other studies as well. In New Mexico, we observe *HER2* overexpression in ~ 25% of GBC cases (*n* = 6/25, unpublished data, see Figs. 4 and 5).

Activation of *KRAS* is identified in a subset of GBCs, with increased activation seen in anomalous junction of the pancreaticobiliary duct (AJPBD). A study specifically looking at *BRAF*, which is a key effector of the oncologic activity of *KRAS*, found *BRAF* mutations in 33% of GBCs studied [64]. Additionally, these mutations were found to be mutually exclusive, which has also been noted in other types of cancer. A recent systematic review and meta-analysis of genomic profiling identified 80 candidate gene variants and 173 polymorphisms associated with GBC in total [65]. Although this analysis found most of the studies to be of indeterminate quality due to the small sample sizes and lack of confirmatory testing, the following candidate genes were found to be significant*: XPC, ERCC2, MSH2, OGG1, XRCC1, EGF, KRAS Gln25His, NAT2, GSTT1, ESR1, and CYP7A1* [65]. The meta-analysis found only the *TP53* mutational changes to be significantly associated with GBC susceptibility [65].

b. Chromosome level changes: Allelic loss of heterozygosity has been observed in GBC at the 1p, 3p, 8p, 9p, 13q, 16q, 17p and 22q locations of the chromosome [48]. Microsatellite instability (MSI) is observed in GBC at rates similar to other GI malignancies [66]. The incidence of MSI in GBC has been reported to be between 0 and 40% with an average of ~ 10% of the GBC patients [66]. In our New Mexican GBC cohort, we observed an elevated tumor mutational burden (TMB) in 8% of our GBC cohort (*n* = 2/25, unpublished data) consistent with observations elsewhere. MSI and TMB are increasingly important as predictive biomarkers of response to immunotherapies. Epigenetic changes have also been linked to GBC pathogenesis. Altered methylation patterns of the *p73, MGMT, MLH1, APC and p16* genes has been identified in 72% of GBCs and 28% of chronic cholecystitis cases [67]. Epigenetic alternations of the DNA repair systems are implicated in the microsatellite instability seen in GBC patients.

In summary, like many other GI malignancies, the diversity of genomic alterations in GBC appears to be high and ill-defined with diverse mutational, copy number, chromosomal and epigenetic changes. The genomic changes in GBC are likely the result of a complex interactions due to the initiating chronic inflammation and environmentally driven factors over a prolonged period. The relative rarity of the tumor is an additional challenge to the detailed genomic level understanding of this form of cancer. Further studies (human and animal models) are urgently required to delineate the precise step-wise progression of genomic changes in GBC.

## Therapy considerations in native American and other rural, and medically underserved GBC populations.

Two-thirds of GBC patients are diagnosed incidentally at the time of surgery for symptomatic cholelithiasis/cholecystitis [68]. Incidental diagnosis of GBC occurs in 0.2–3% of all cholecystectomies for presumed benign disease [69]. The remaining one third of patients present with symptoms from locally advanced disease, including right upper quadrant/epigastric pain, obstructive jaundice, nausea, anorexia and weight loss. Patients presenting with symptomatic disease tend to be locally advanced/metastatic at presentation and 75% are found with unresectable disease [70].

The standard of care for both curative and palliative treatment of GBC with regards to surgery, chemotherapy, and radiation are summarized in Table 1. The treatment guidelines are broadly applicable to Native American patients and other minority, rural, and medically underserved patient groups. Yet, Native American communities have poorer cancer outcomes, suboptimal cancer screening, and high-risk cancer behaviors [71]. Native American cancer patients are less likely to undergo recommended cancer surgeries, adjuvant chemotherapy, and radiation therapy compared to the non-Native populations. Due to these factors, Native Americans have the worst cancer survival rates of any US ethnic group in general [72]. Despite these recognized health disparities, Native American patients with GBC did not demonstrate a significant differences in stage at diagnosis or survival compared to non-Native American populations [73]. This may be due to the advanced stage and poor prognosis associated with GBC in general, regardless of access to healthcare. There is a paucity of GBC treatment data in Native American populations. Access to specialized surgical care is critical in GBC management. Markin et al. evaluated whether operative outcomes contribute to poor long-term survival among Native American patients with cancer, including a small subset with GBC [74]. Their analysis demonstrated that Native American patients were younger, more likely to undergo cancer surgery at rural hospitals, and more likely to be admitted for non-elective procedures and had more comorbidities compared to non-Hispanic white patients of similar ages (all, *P* < .05). Native American patients had comparable inpatient mortality and length of stay. The authors suggest that future research should focus on other cancer care delivery factors that may contribute to the poor long-term survival of Native American patients with cancer, including delivery of perioperative therapies.

**Table 1:** A summary of the current standard of care for curative and palliative treatment of gallbladder cancers in routine clinical practice

Primary tumor	Stage	Surgical approach	Chemotherapy	Radiation therapy
T1a tumors	Stage I	simple cholecystectomy	Not indicated	Not indicated
T1b – T3 tumors	Stage I-III	Radical cholecystectomy: hepatic resection and lymph node dissection with or without common bile duct resection and reconstructive hepaticojejunostomy.	Consensus-based guidelines for adjuvant therapy after resection of bile duct cancer are available from two expert groups: • National Comprehenisve Cancer Network (NCCN): Consider adjuvant fluoropyrimidine-based chemoradiotherapy, or chemotherapy alone with a single-agent fluoropyrimidine or gemcitabine should be considered after resection for all except T1 N0 tumors. • European Society of Medical Oncology (ESMO): Consider postoperative chemoradiotherapy after complete surgical resection of any-stage disease.	
			Adjuvant therapy options/Supporting clinical trials	
			BILCAP trial (phase III) *Population*: 447 patients with resected cholangiocarcinoma or GBC (*n* = 79) *Intervention*: Capecitabine 1250 mg/m2 BID days 1–14 every 21 days × 8 cycles versus placebo *Outcome*: Clinically but not statistically significant improvement in overall survival (OS) with capecitabine by intent to treat analysis (median OS 51 versus 36 months, HR 0.81, 95% CI 0.63–1.06, *p* = 0.097).	SWOG S0809 (phase II) *Population*: 79 patients with resected extrahepatic biliary cancer (*n* = 54) or GBC (*n* = 25) *Intervention*: Gemcitabine 1000 mg/m2 day 1 and day 8 + capecitabine 1500 mg/m2/day days 1–14 every 21 days × 4 cycles followed by concomitant capecitabine 1330 mg/m2/day and with RT (45 Gy to regional lymphatics, 54 to 59.4 Gy to tumor bed). *Outcome*: Median overall survival 35 months.
			PRODIGE 12-ACCORD 18 trial (phase III) *Population*: 196 patients with resected biliary tract cancer including GBC (*n* = 37) *Intervention*: Gemcitabine 1000 mg/m2 day 1 + oxaliplatin 85 mg/m2 day 2 every 21 days × 12 cycles versus surveillance *Outcome*: Adjuvant gemcitabine + oxaliplatin did not significantly improve relapse-free survival, the primary endpoint (at four years, 39 versus 33%; HR 0.83, 95% CI 0.58–1.19, *p* = 0.31).	
			ACTICCA-1 trial (phase III) (ongoing, NCT02170090) *Population*: Open to patients with resected cholangiocarcinoma or GBC *Intervention*: Cisplatin 25 mg/m2 + Gemcitabine 1000 mg/m2 days 1 and 8 every 21 days × 6 cycles versus Capecitabine 1250 mg/m2 BID days 1–14 every 21 days × 8 cycles versus observation *Outcome*: pending	
T4	Stage IV	Not indicated: Although biliary or intestinal bypass can be considered, percutaneous or endoscopic approaches are generally preferred, given the limited median survival in patients with advanced disease.	Palliative therapy option/Supporting clinical trials	
Any T, with distant metastatic disease	Stage IV		*First line chemotherapy:* ABC-02 trial Phase III *Population*: 410 patients with locally advanced or metastatic bile duct, gallbladder (*n* = 148), or ampullary cancer. *Intervention*: Cisplatin 25 mg/m2 + gemcitabine 1000 mg/m2 on days 1 and 8, every 21 days versus single agent gemcitabine 1000 mg/m2 days 1, 8, 15 every 28 days. *Outcome*: Median overall survival was significantly greater with combination therapy (11.7 versus 8.1 months, (*P* < 0.001).). Rate of tumor control among patients in the cisplatin-gemcitabine group was also significantly increased (81.4% vs. 71.8%, *P* = 0.049).	In select patients with unresectable disease and initial good systemic control, radiation therapy can be discussed on a case by case basis.
			*Second line chemotherapy:* No regimen can be considered standard after failure of an initial gemcitabine-based regimen. However, in patients who retain a good performance status oxaliplatin-based regimen, 5-FU/capecitabine, taxanes, or irinotecan based therapy may be considered.	

The role of adjuvant therapy in GBC remains ill-defined, though it is recommended by both the National Comprehensive Cancer Network (NCCN) and the European Society of Medical Oncology (ESMO) for Stage I-III disease following surgery with curative intent (Table 1). Oral capecitabine, as administered in the BILCAP trial, is a particularly appealing adjuvant therapy option for patients living in a rural setting far away from major treatment centers, as is the case for many New Mexican GBC patients. Adjuvant radiation therapy access remains a unique challenge for rural cancer patients. Athas et al. evaluated 1122 cases of localized breast cancer using the New Mexico NCI SEER Tumor Registry (NMTR) [75]. After adjusting for age, the likelihood of receiving radiation therapy following breast conserving surgery decreased significantly with increased travel distance to the nearest radiation oncology facility, with 51% of women living 75 miles or more from the closest facility receiving radiation compared with 82% residing within 50 miles travel distance. These findings highlight the difficulty rural cancer patients have accessing specialized cancer care including a New Mexico -centric cancer such as GBC.

Rural and underserved cancer patient populations also experience disparities in supportive palliative care services. This was demonstrated in a SEER analysis of Native American and White patients dying of breast, cervix, colorectal, kidney, lung, pancreas, prostate cancer, or stomach cancer from 2003 to 2009 [76]. In this analysis, a lower proportion of Native Americans enrolled in hospice compared to White patients (54% vs 65%, respectively; *P* < .0001). While the proportion of White patients who used hospice services in the last 6 months of life increased from 61% in 2003 to 68% in 2009 (*P* < .0001), the proportion of American Indian patients using hospice care remained unchanged (*P* = .57) and remained below that of their Caucasian counterparts throughout the study [76]. Telemedicine may serve as a potential solution to address these disparities. For example, Project ECHO (Extension for Community Healthcare Outcomes) originating at University of New Mexico, uses teleconferencing technology to support and train healthcare providers (HCPs) in remote rural locations. ECHO has been successfully used to provide education and support to community hospice nurses [77, 78]. Our center is currently piloting the use of Project ECHO to facilitate complex cancer care and access to clinical trials to community oncology patients within the state of New Mexico. GBC, a disease requiring access to multidisciplinary specialized care including medical and surgical oncology, radiation oncology, interventional radiology and gastroenterology, as well as palliative care, is a disease that will benefit from such an intervention.

## Prevention strategies in gallbladder cancers

Given the fact that GBC is most frequently diagnosed when the disease is advanced and no longer curable, effective screening modalities are of critical importance. A successful screening strategy will require the identification of patients at significantly high risk for GBC based on the risk factors discussed above. Some practitioners recommend the use of prophylactic cholecystectomy in patients with silent gallstones in areas of high prevalence of disease. Empiric evidence suggests increased rates of cholecystectomies can have a significant effect on the rate of GBC. In India where there is a known high prevalence of GBC, it was found that 1 case of GBC could be prevented for every 67 laparoscopic cholecystectomies. Comparatively, in regions where the GBC is relatively low (e.g., continental US), one case of GBC is preventable for every 769 cholecystectomies [79]. In response to abnormally high incidence of GBC, the government of Chile instituted a program of prophylactic cholecystectomy in 2006 in people aged 35–49 years with gallstones which is currently underway. Yet, the health care costs associated with prospective surgical interventions (e.g., cholecystectomies) represent a significant financial burden in low-GDP countries like India and Chile, potentially limiting the scope of such approaches. Similarly, the risks associated with surgical interventions are significant. Lastly, given the inciting role for inflammation in this disease, NSAIDs may play a role in chemoprevention and requires further research.

## Future directions in the treatment of GBC

Guideline based palliative systemic therapy for GBC is limited to cytotoxic chemotherapy (see Table 1). As with other malignancies, multiple efforts are underway globally to identify targeted agents for the treatment of GBC, though none are established as standard of care in routine clinical practice (see Table 2). Many of the clinical trials group biliary tract cancers together (intra-hepatic cholangiocarcinoma, extra-hepatic cholangiocarcinoma, and GBC), making stand-alone analysis of GBC difficult. We briefly describe some of the promising approaches below for GBC.

**Table 2:** Current clinical trials in progress for targeted therapeutic options for gallbladder cancers (data from www.clinicaltrials.gov)

Agent	Target	Regimen	Phase	Institution	NCI Number
Cetuximab, Trastuzumab, Gefitinib, Lapatinib, Everolimus, Sorafenib, Crizotinib	EGFR, HER2, mTOR, VEGF, ALK/ROS1, PDL-1	GEMOX + targeted therapy per proteomic/genetic profiling	II	Xinhua Hospital, Shanghai	NCT02836847
Durvalumab	PDL-1	Guadecitabine + Durvalumab	I	University of Southern California	NCT03257761
Pazopanib	cKIT, FGFR, VEGFR	Gemcitabine + Pazobanib	II	Hellenic Cooperative Oncology Group	NCT01855724
Pembrolizumab	PD-1	Pembrolizumab + Cisplatin + Gemcitabine	II	European Organization for Research and Treatment of Cancer	NCT03260712
Selumetinib	MEK1, MEK2	Selumetinib + Cisplatin + Gemcitabine	II	University Health Network, Toronto	NCT02151084
ADH-1	N-Cadherin	ADH-1 + Cisplatin + Gemcitabine	I	University of Nebraska	NCT01825603
Canlisib	PI3K	Copanlisib + Cisplatin + Gemcitabine	II	H. Lee Moffitt Cancer Center and Research Institute	NCT02631590
Merestinib	MET	Merestinib + Cisplatin + Gemcitabine	I	Eli Lilly and Company	NCT03027284
Regorafenib	VEGF	Regorafenib	II	H. Lee Moffitt Cancer Center and Research Institute	NCT02115542

### HER2 and EGFR targeting therapies

Targeting the *HER2/EGFR* family pathway has become increasingly attractive in a variety of gastrointestinal malignancies. The HER2 protein has been shown to be variably amplified in 16–64% of GBC [80, 81]. Similarly, EGFR amplifications and mutations have been described in 6 and 13.6%–15% respectively [82]. Unfortunately, early clinical trials to date have not demonstrated a significant benefit in targeting these pathways in isolation. In in-vitro studies with lapatinib, a dual tyrosine kinase inhibitor which interrupts both the *HER2* and *EGFR* pathways, demonstrated a synergistic anti-tumor effect on GBC cell lines when combined with gemcitabine [83]. These promising pre-clinical results led to two phase II trials of single agent lapatinib in biliary tract cancers (including GBC). These trials were ultimately negative, however the study population was not selected for relevant *HER2* amplification [82]. Another second case series followed nine patients treated with *HER2* directed therapy (trastuzumab, lapatinib, or pertuzumab) either alone or in combination with chemotherapy demonstrating one complete response, four partial responses, and three cases with stable disease [84]. One patient treated with lapatinib alone had mixed response, indicating a possible role of *HER2* monotherapy. A phase II trial investigating trastuzumab in unresectable biliary tract cancer was closed prematurely due to difficulty with enrollment and lack of response signal (NCT00478140).

With regards to *EGFR*, a phase III trial evaluated the efficacy of erlotinib, a tyrosine kinase inhibitor targeting *EFGR*, combined with gemcitabine and oxaliplatin, in patients with advanced biliary tract cancer has been completed. Progression free survival was 5.8 months in the erlotinib group compared with 4.2 months, but overall survival was the same for both groups and no benefit was noted in GBC patients [85]. Several phase II trials investigating panitumumab, a monoclonal antibody targeted to *EGFR*, combined with chemotherapy in *KRAS* wild-type patients with biliary tract cancer have demonstrated mixed results, with the largest randomized trial showing no difference in survival [82]. Targeted inhibitors in combination with regular chemotherapy appears to show promise for future GBC treatments.

### Other therapeutic targets

Several clinical trials have investigated the use of *VEGF* inhibition in GBC. In a phase II trial in all biliary tract cancers, bevacizumab, a monoclonal antibody that inhibits *VEGF-A*, combined with gemcitabine and oxaliplatin, demonstrated a 6 month PFS of 63%, which was below the target rate of 70% [86]. Another phase II study using cediranib, an oral *VEGFR1, VEGFR2, and VEGF3* tyrosine kinase inhibitor, similarly failed to meet its primary endpoint of improvement in median PFS [87]. Studies investigating antiangiogenic tyrosine kinase inhibitors sorafenib and sunitinib have been similarly disappointing [82]. Other pathways of interest for targeting purposes in GBC include *APC, BRAF, KRAS, and P53*. Similarly, immunotherapy has shown promising signals in GBC. In a study of biliary tract cancer patients, 42% of 89 patients were found to be PD-L1 positive [82]. Numerous ongoing clinical trials utilizing immune checkpoint inhibitors include subsets of biliary tract cancer. Of particular interest is a study at Xinhua Hospital in Shanghai that seeks to assign targeted therapy to biliary tract cancer patients according to relevant mutations (NCT02836847). Results from this and several other trials are pending. Table 2 provides a summary of ongoing clinical trials with targeted therapies for GBC. There is much excitement for the potential of targeted drug and immunotherapy based approaches in GBC treatment, through much pre-clinical and clinical work remains to be done to demonstrate the enhanced efficacy of these newer therapeutic approaches.

## Conclusions

GBC is a relatively rare disease with a poor clinical prognosis and with a unique geographic distribution and risk factor profile. In the United States, Native American populations in the Southwest are uniquely impacted. The elevated incidence of gallstones among Native Americans is likely to be the major driving factor of increased of GBC in this population. Unfortunately, treatment options for this disease are still quite limited. Surgical resections, cytotoxic chemotherapy and radiation are the mainstay of GBC treatments, all with modest survival benefit. Research efforts are currently underway to understand the molecular pathogenesis of GBC, which may aid in identifying targeted therapeutic approaches. The difficulty in treating GBC is in part due to the relative rarity of the tumor and the lack of robust basic research efforts. The lack of readily available animal models of GBC also presents a significant hurdle to our understanding. Lastly, many GBC patients present at an advanced stage with a poor performance status, rendering them ineligible for clinical trial participation. Given the high mortality and lack of therapeutic options, GBC patients will benefit from enhanced efforts at developing pre-clinical research with subsequent translation to therapeutic clinical trials with the goal of improving survival outcomes.

## Abbreviation

GBC: Gallbladder Cancer
